# ROS Production via P2Y_1_-PKC-NOX2 Is Triggered by Extracellular ATP after Electrical Stimulation of Skeletal Muscle Cells

**DOI:** 10.1371/journal.pone.0129882

**Published:** 2015-06-08

**Authors:** Alexis Díaz-Vegas, Cristian A. Campos, Ariel Contreras-Ferrat, Mariana Casas, Sonja Buvinic, Enrique Jaimovich, Alejandra Espinosa

**Affiliations:** 1 Departamento de Tecnología Médica, Facultad de Medicina, Universidad de Chile, Santiago, Chile; 2 Centro de Estudios Moleculares de la Célula, ICBM, Facultad de Medicina, Universidad de Chile, Santiago, Chile; 3 Instituto de Investigación en Ciencias Odontológicas, Facultad de Odontología, Universidad de Chile, Santiago, Chile; 4 Programa de Fisiología y Biofísica, Instituto de Ciencias Biomédicas, Facultad de Medicina, Universidad de Chile, Santiago, Chile; University of Debrecen, HUNGARY

## Abstract

During exercise, skeletal muscle produces reactive oxygen species (ROS) via NADPH oxidase (NOX2) while inducing cellular adaptations associated with contractile activity. The signals involved in this mechanism are still a matter of study. ATP is released from skeletal muscle during electrical stimulation and can autocrinely signal through purinergic receptors; we searched for an influence of this signal in ROS production. The aim of this work was to characterize ROS production induced by electrical stimulation and extracellular ATP. ROS production was measured using two alternative probes; chloromethyl-2,7- dichlorodihydrofluorescein diacetate or electroporation to express the hydrogen peroxide-sensitive protein Hyper. Electrical stimulation (ES) triggered a transient ROS increase in muscle fibers which was mimicked by extracellular ATP and was prevented by both carbenoxolone and suramin; antagonists of pannexin channel and purinergic receptors respectively. In addition, transient ROS increase was prevented by apyrase, an ecto-nucleotidase. MRS2365, a P2Y_1_ receptor agonist, induced a large signal while UTPyS (P2Y_2_ agonist) elicited a much smaller signal, similar to the one seen when using ATP plus MRS2179, an antagonist of P2Y_1_. Protein kinase C (PKC) inhibitors also blocked ES-induced ROS production. Our results indicate that physiological levels of electrical stimulation induce ROS production in skeletal muscle cells through release of extracellular ATP and activation of P2Y1 receptors. Use of selective NOX2 and PKC inhibitors suggests that ROS production induced by ES or extracellular ATP is mediated by NOX2 activated by PKC.

## Introduction

During exercise, several pathways are activated in skeletal muscle in order to maintain cellular homeostasis [1]. Skeletal muscle responds to exercise or electrical stimuli with an increased generation of reactive oxygen species (ROS) [2]. ROS are produced during cell metabolism from different sources, among them xanthine oxidase, mitochondria and NADPH oxidase (NOX) [3]. NADPH oxidases are proteins that transfer electrons across biological membranes. In general, the electron acceptor is oxygen and the product of the electron transfer reaction is superoxide (O_2_^-^) which is then converted to hydrogen peroxide (H_2_O_2_) by the enzyme superoxide dismutase (SOD) [4]. NOX family members are transmembrane proteins. The phagocyte NADPH oxidase 2 (NOX2) was the first identified and is the best studied member of the NOX family. Depending of the type of cell, in resting conditions gp91^phox^ and p22^phox^ are found primarily in the plasma membrane. Upon activation, the movement of cytoplasmic subunits, p67^phox^, p47^phox^, p40^phox^ and Rac GTPase from the cytoplasm to the membrane form the active NOX2 enzyme complex [4]. NOX2 can be activated by several mechanisms like p47^phox^ phosphorylation by PKC or by PI3K [5]. Skeletal muscle cells express NOX2 [6] and several authors suggest that this is one of the main sources of ROS during muscle contraction or electrical stimuli [7, 8], in addition, NOX protein subunits were detected in transverse tubules and triads isolated from rabbit skeletal muscle but not in sarcoplasmic reticulum vesicles [9], moreover, electrical stimulation induces NOX2 activation in skeletal muscle cells [7]; the mechanism of activation, however, is not fully understood.

ROS can modulate several pathways such as mitochondrial biogenesis, cell proliferation, muscle plasticity, phosphatase and kinase activities and antioxidant expression to maintain cellular homeostasis [5, 10–14]. For example, in myotubes, ROS stimulate ERK, CREB, early genes and glucose uptake induced by insulin [7, 15].

We have previously studied depolarization-induced calcium signals in skeletal muscle cells, describing a fast calcium transient involved in excitation-contraction coupling, and a slow, nuclei-associated calcium transient unrelated to contraction [16, 17]. The slow calcium signal is related with IP_3_R activation [18, 19] and depends on ATP released from the stimulated muscle cells [20]. ATP signals in skeletal muscle through P2Y purinergic receptors [20]. P2Y are G-protein–coupled receptors that typically signal through the β**γ** subunits to activate phosphatidylinositol 3-kinase-**γ** (PI3K**γ**) and PKC [19, 21]. We hypothesized that ATP extruded from the muscle fiber increases ROS production via PKC-NOX2. Our results indicate that both electrical stimulation and extracellular ATP induced ROS production in skeletal muscle cells, at least partly through NOX2 activation via P2Y_1_-PKC.

## Materials and Methods

### Isolation of adult fibers

We used C57/BL6J mice (6–8 weeks old) obtained from the Animal Facility at the Faculty of Medicine, University of Chile. Mice were sacrificed by exposure to isofluorane (5%) followed by cervical dislocation. Fibers were isolated from *flexor digitorum brevis* (FDB) muscle after enzyme digestion with type 2 collagenase (90min with 400U/ml; Worthington Biochemicals Corp., Lakewood, NJ, USA), and mechanical dissociation with fire-polished Pasteur pipettes, as described previously [17]. All the procedures performed in this work were approved by the Bioethics Committee of the Faculty of Medicine, University of Chile.

### ROS production

ROS generation in skeletal muscle cells was evaluated using chloromethyl-2,7- dichlorodihydrofluorescein diacetate (DCF) probe (Eugene, OR). Muscle fibers were cultured on glass coverslips and incubated with 5 μM DCF during 15 min at 37°C. The cells were washed with PBS (137mM NaCl, 2.7mM KCl, 4.3mM Na_2_HPO_4_, 1.47 mM KH_2_PO_4_). Cultures on coverslip dishes were transferred to the confocal microscope (Carl Zeiss Pascal 5, LSM). DCF fluorescence was detected using excitation-emission at λ488/λ510–540 nm. In all measurements, a control with laser excitation only was performed. The laser illumination was kept at a minimum (stand by position, 0.1–0.3% potency). All experiments were conducted in Krebs buffer with or without calcium (Krebs with calcium: 145 mM NaCl, 5 mM KCl, 1 mM CaCl_2_, 1 mM MgCl_2_, 5.6 mM glucose, 10 mM HEPES, pH 7.4; Krebs without calcium: 145 mM NaCl, 5mM KCl, 3.6 mM MgCl_2_, 10 mM HEPES, 2mMEGTA, 5.6 mM glucose). The results were expressed as (ΔF/F_0_)*100 and fluorescence signals were processed as previously described [22], the fluorescence profiles were fit by linear regression. The DCF fluorescence change was normalized to the control to allow comparison of rates after the stimulus. NOX-specific inhibitor gp91dsTAT (5μM) (*YGRKKRRQRRRCSTRIRRQL—NH2)* [23] or apocynin (50μM) were used to verify the source of ROS. PnX1 inhibitor carbenoxolone (CBX) (5μM), purinergic antagonist suramin (10μM) were used to verify ATP release and purinergic receptor in NOX2 activation, PKC general inhibitor BIM (0.5μM) was used to determine the signal pathway. 25 μM N-benzyl-p-toluene sulphonamide (BTS, from Sigma-Aldrich Co., St Louis, MO, USA) was used in all experiments as a fiber contraction inhibitor.

### Fibers Transfection and H_2_O_2_ Measurement

H_2_O_2_ production was evaluated using a plasmid that encodes for HyPer protein. Muscle fibers were cultured on glass coverslips and transfected using Lipofectamin 2000 (Invitrogen, Carlsbad, CA, USA) for 2 h (1 μg DNA/3 μL) during collagenase digestion of FDB muscle. H_2_O_2_ was determined 24h post transfection, as described previously [24]. HyPer imaging was achieved using an inverted Olympus IX81 microscope with a 40x objective (numerical aperture, N.A. 1.3). HyPer fluorescence was detected using an excitation/emission wavelength λexc1−λexc2/λem = 420−490/520 nm. The ratio between the signals excited with 490 and 420 nm was used to determine the presence of H_2_O_2_, HyPer has a 420 nm excitation peak that decreases in proportion to the increase at 490 nm [25]. Fluorescence emitted at 520 nm was shown. Each experiment was performed alongside the effect of laser excitation alone. Noise in the images was removed using Image J Filters (http://rsbweb.nih.gov/ij/). All experiment were conducted in Krebs buffer (145 mM NaCl, 5 mM KCl, 1 mM CaCl_2_, 1 mM MgCl_2_, 5.6 mM glucose, 10 mM HEPES, pH 7.4). We used MRS2179 (1μM), MRS2365 (20μM) and UTPγS (20μM) to verify purinergic receptor participation in the process. All reagents were obtained from Sigma-Aldrich Co., St Louis, MO, USA.

### Muscle fibers stimulation

Adult muscle fibers were stimulated with either, electrical stimulation (ES) or extracellular ATP. Electrical stimulation was a single sequence of 270 square pulses of 0.3 ms duration at 20 Hz or 90 Hz (lasting 13.5 or 3s) using electrodes that consist of a row of six platinum wires intercalated 0.5 cm apart with alternate polarity across a circular plastic holder that fits in the dish, as described previously [26].

### Data analysis

All values are expressed as means ± standard error of the mean (S.E.M.) from at least three different experiment. The significance of differences was evaluated using Student’s t-test for paired data and one-way ANOVA followed by Dunnett’s post-test for multiple comparisons or Bonferroni’s post-test for multiple paired comparisons. P<0.05 was considered to be statistically significant. Graph- Pad PRISM 5.03 software (GraphPad Software Inc, LA, USA) was used for data fitting.

## Results

### Electrical stimulation and extracellular ATP increase ROS production in skeletal muscle fibers

**Fig 1:**
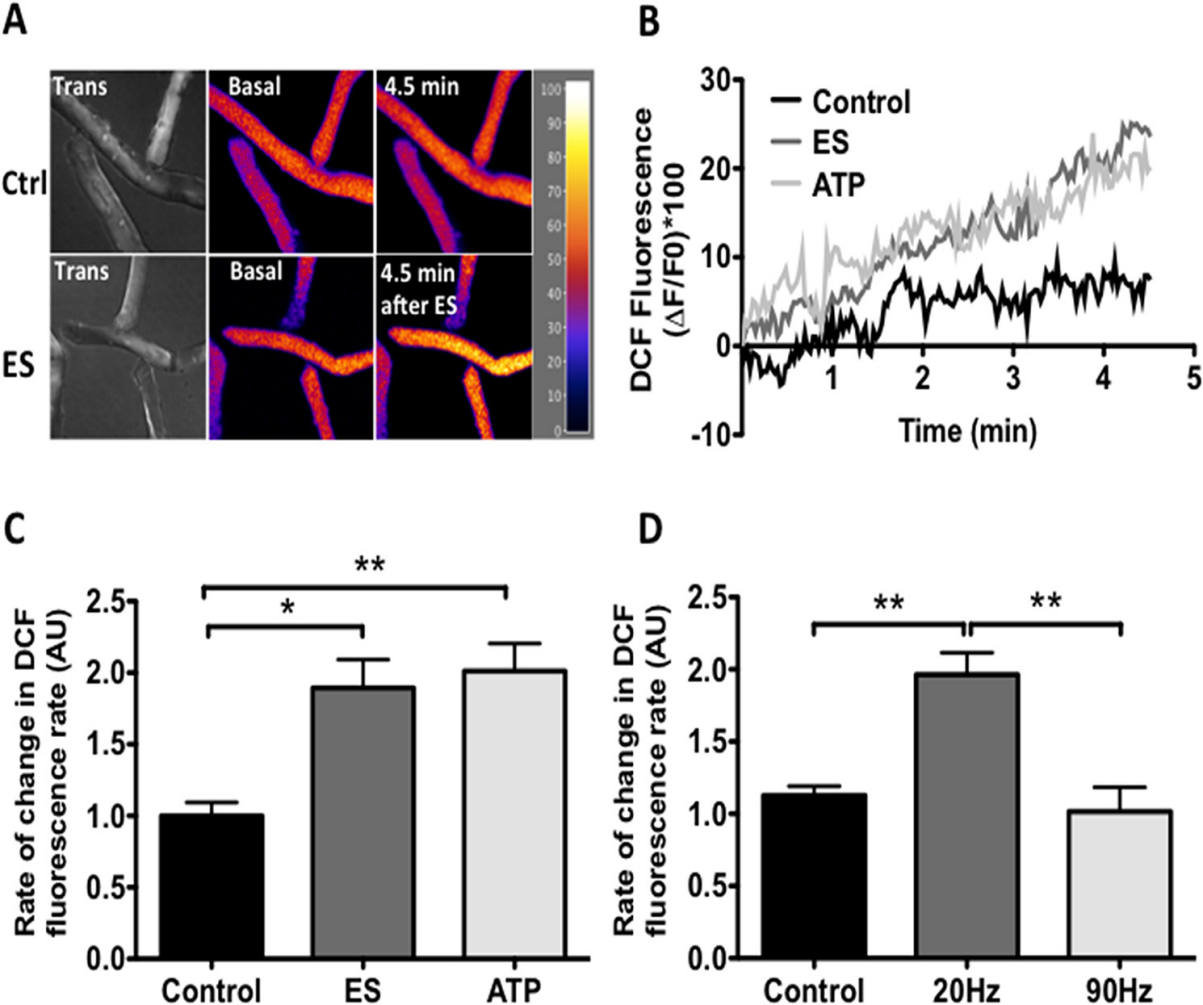
Electrical stimulation and exogenous ATP induced increase of ROS production. Muscle fibers were isolated, load with DCF (30min) and stimulated with electrical stimulation (ES) (20Hz) or exogenous ATP (10μM). A. Representative image in pseudo-color of a cell loaded with DCF in control condition (upper panel) and stimulated with ES (lower panel). B, representative traces of DCF fluorescence under control or stimulated with ES or exogenous ATP. C, muscle cells were stimulated with ES or ATP and the slope of fluorescence is shows (*see* *material and method*). D, muscle cells were stimulated with ES at 20Hz or 90Hz and the slope of fluorescence is show (*see* *Materials and Methods*) (n = 4, *p<0.05, **p<0.01).

ES induces an increase in extracellular ATP [26] and we studied ROS production using the DCF probe. The probe fluorescence is clearly higher minutes after electrical stimulation compared with the unstimulated control (Fig 1A). Because autoxidation of the probe when exposed to light scan occurs, generating artifactual signals, the slopes of the fluorescence increase after stimulation were quantified and normalized against the control condition, as described previously by other group [27, 28]. Thus, the ES induced a sustained fluorescence increase with a slope of 1.89 fold over control condition (Fig 1B and 1C). In addition, the exogenous administration of ATP (10μM) increased the fluorescence of DCF similarly, in magnitude, timing and speed, compared to ES (2.01 fold over control) (Fig 1B and 1C). Finally, we have previously reported that ATP release from muscle cells is frequency-dependent, ATP is released from muscle fibers at low frequency but not at high frequency [26]; in agreement with those results, we observed an increase of ROS production at 20Hz but not at 90Hz, suggesting that ROS increase during muscle activity is dependent on release of extracellular ATP (Fig 1D).

### ROS production induced by electrical stimulus of adult skeletal muscle fibers is dependent on ATP release and purinergic receptor P2Y_1_

**Fig 2:**
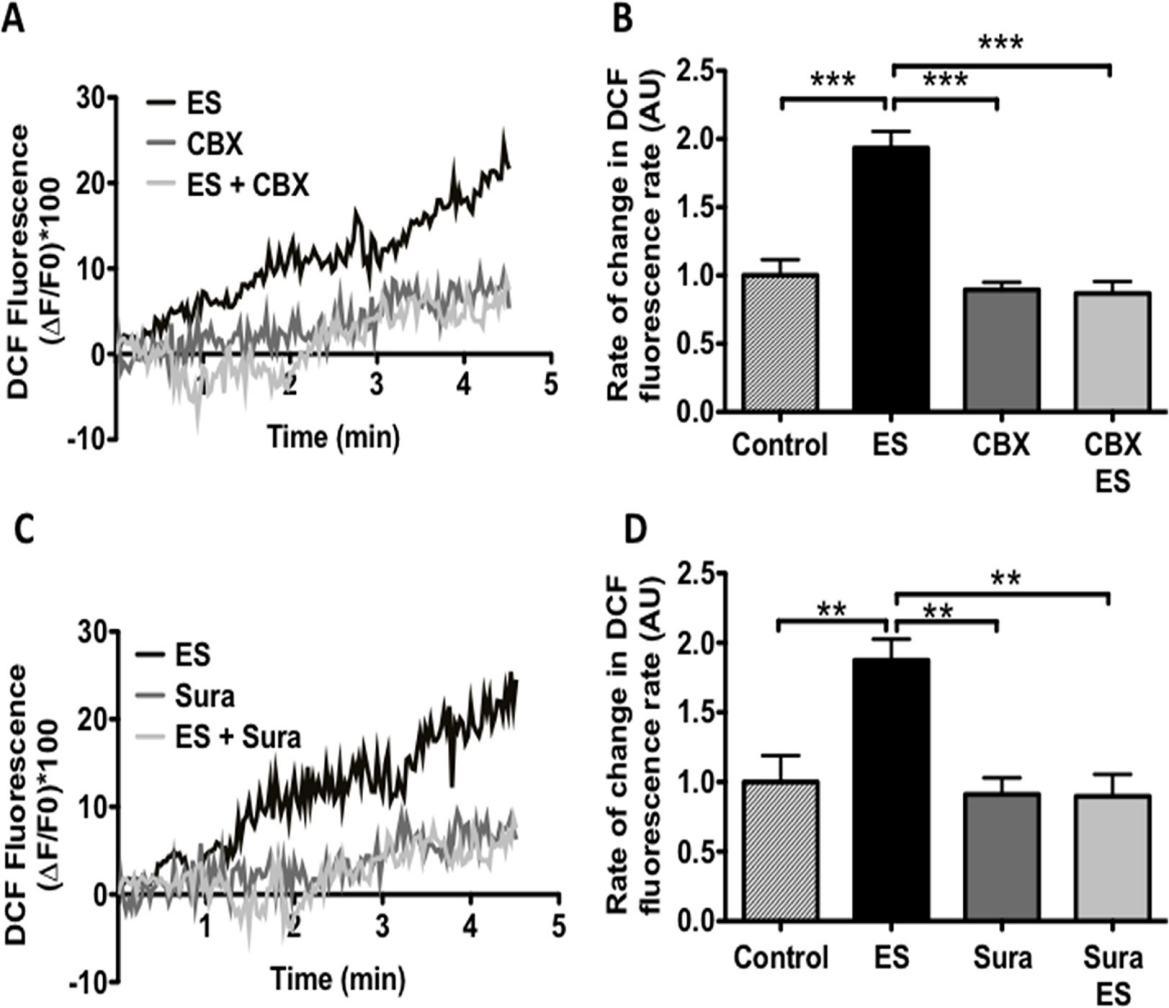
Electrical stimulation induced ROS production via ATP release and purinergic receptor. Muscle fibers were isolated, load with DCF fluorescence (30 min) under control or stimulated, in the absence or presence of different inhibitors (30 min of incubation). A, representative traces of DCF fluorescence under control or stimulated, in absence or presence of CBX (10μM). B, slope of fluorescence from muscle cells stimulated with ES (*see* *material and method*), in the absence or presence of CBX. C, representative traces of DCF fluorescence under control or stimulated, in the absence or presence of suramin (10μM). D, slope of fluorescence from muscle cells stimulated with ES, in the absence or presence of suramin. (n = 6), **p<0.01, ***p<0.001.

We evaluated whether ATP extrusion to the extracellular medium after electrical stimulation is capable to increase ROS production in adult fibers. Accordingly, the increase in ROS production induced by ES was prevented when fibers were pre-incubated with CBX, an inhibitor of PnX channel (Fig 2A and 2B). Furthermore, pretreatment with the purinergic receptor antagonist suramin inhibited electrical stimulus-dependent ROS production (Fig 2C and 2D).

**Fig 3:**
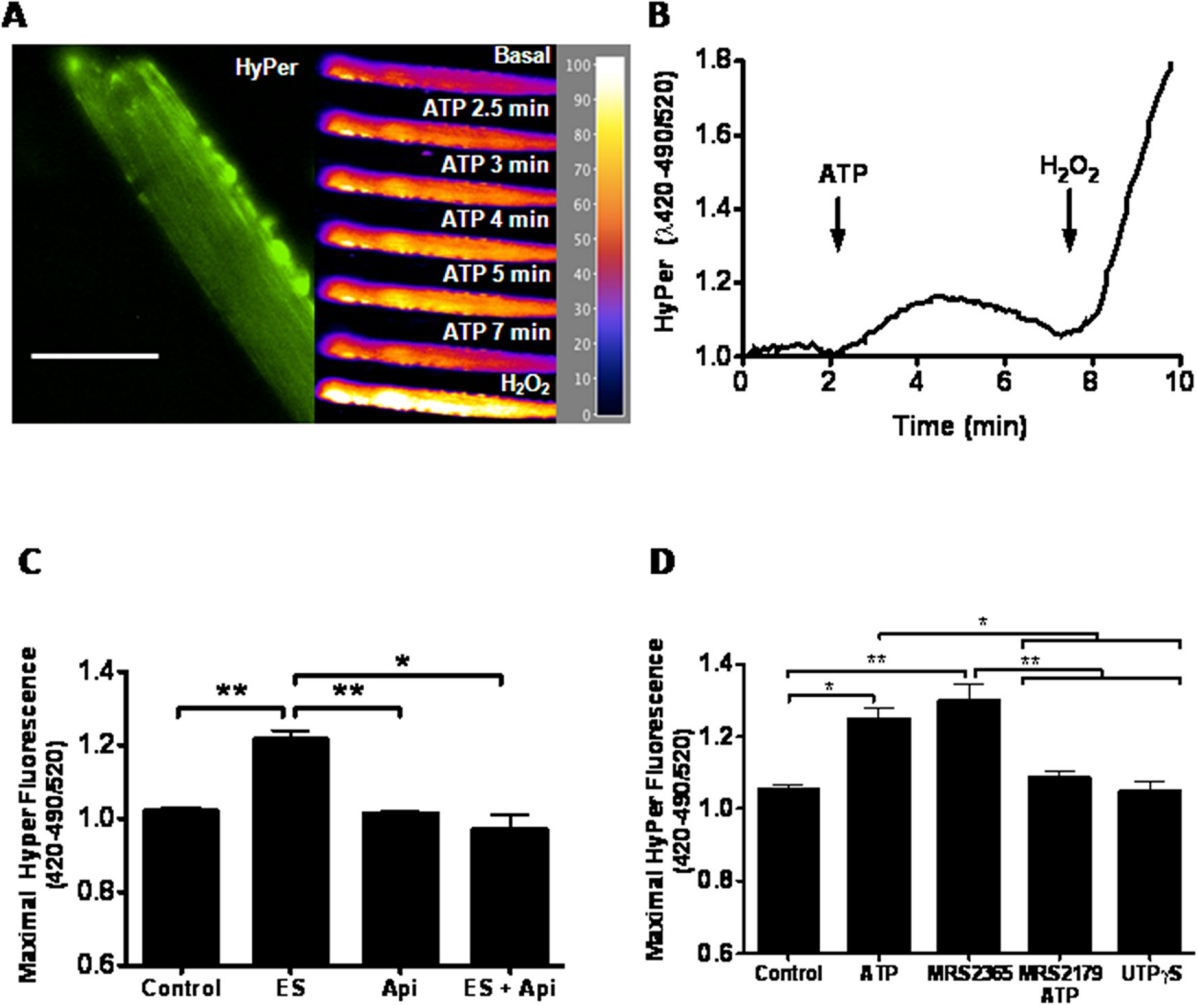
Exogenous ATP increases H_2_O_2_ production via P2Y_1_ purinergic receptor. Muscle fibers were isolated and transfected with HyPer plasmid, 24h post transfection the cells were stimulated. A, H_2_O_2_ generation was measured before and after ATP (10μM) addition. Left panel shows a representative image of Hyper transfection, right panel image fluorescence in pseudo-color. The scale bar represents 50μm. B, kinetics of extracellular ATP-induced H_2_O_2._ C, muscle fibers were transfected with with HyPer plasmid and stimulated with ES in absence or presence of apyrase (2U/ml). Maximal fluorescence was plotted D, C. Effect of Mrs2365, Mrs2179, UTPγS or exogenous ATP, maximal fluorescence was plotted (n = 4), *p<0.05, **p<0.01.

To confirm that extracellular ATP increases the specific production of H_2_O_2_, muscle fibers were transfected with the plasmid encoding the cytoplasmic fluorescent protein Hyper [25]; successful transfection of a fiber with the GFP containing probe can be seen in Fig 3A (left panel). ATP caused a transient increase in Hyper fluorescence of about 20% above basal, which reached a maximum after 3min and returned to resting condition 7 min post stimulation (Fig 3A, right panel and 3B). Similarly, electrical stimulation at 20 Hz induced HyPer fluorescence increase, no increment in H_2_O_2_ production was detected after electrical stimulation in fibers pre-incubated for 30 min with 2 U/ml apyrase, a nucleotidase that metabolizes extracellular ATP to AMP (Fig 3C). Considering that P2Y receptors appear to be involved in this H_2_O_2_ increase, we tried to identify the particular isoform that participate in this process. Recently our laboratory showed that skeletal muscle mainly expresses P2Y_1_ and P2Y_2_ purinergic receptors [29]. So we used agonists and antagonist of these receptors [30]. As the maximal fluorescence was detected 3 min post electrical stimulation, we used this time for all measurements. We observed a similar H_2_O_2_ increase when using exogenous ATP or Mrs2365 (P2Y_1_ agonist) (18 ± 2% and 23 ± 4% respectively), while UTPγS (P2Y_2_ and P2Y_4_ agonist) and Mrs2179 (P2Y_1_ antagonist) plus ATP did not cause any H_2_O_2_ increase (Fig 3D). Finally, when the experiments were performed in the absence of external calcium (data not shown), similar results for electrical stimulation and ATP addition were obtained, suggesting that calcium entry, either through P2X purinergic receptors or through other pathways is not playing a relevant role in this effect. These results suggest that ATP released from the muscle fibers during ES increase H_2_O_2_ production in adult muscle; this effect appears to be mediated by the P2Y_1_ purinergic receptor.

### ROS production induced by extracellular ATP is dependent on NADPH oxidase 2 enzyme (NOX2)

**Fig 4:**
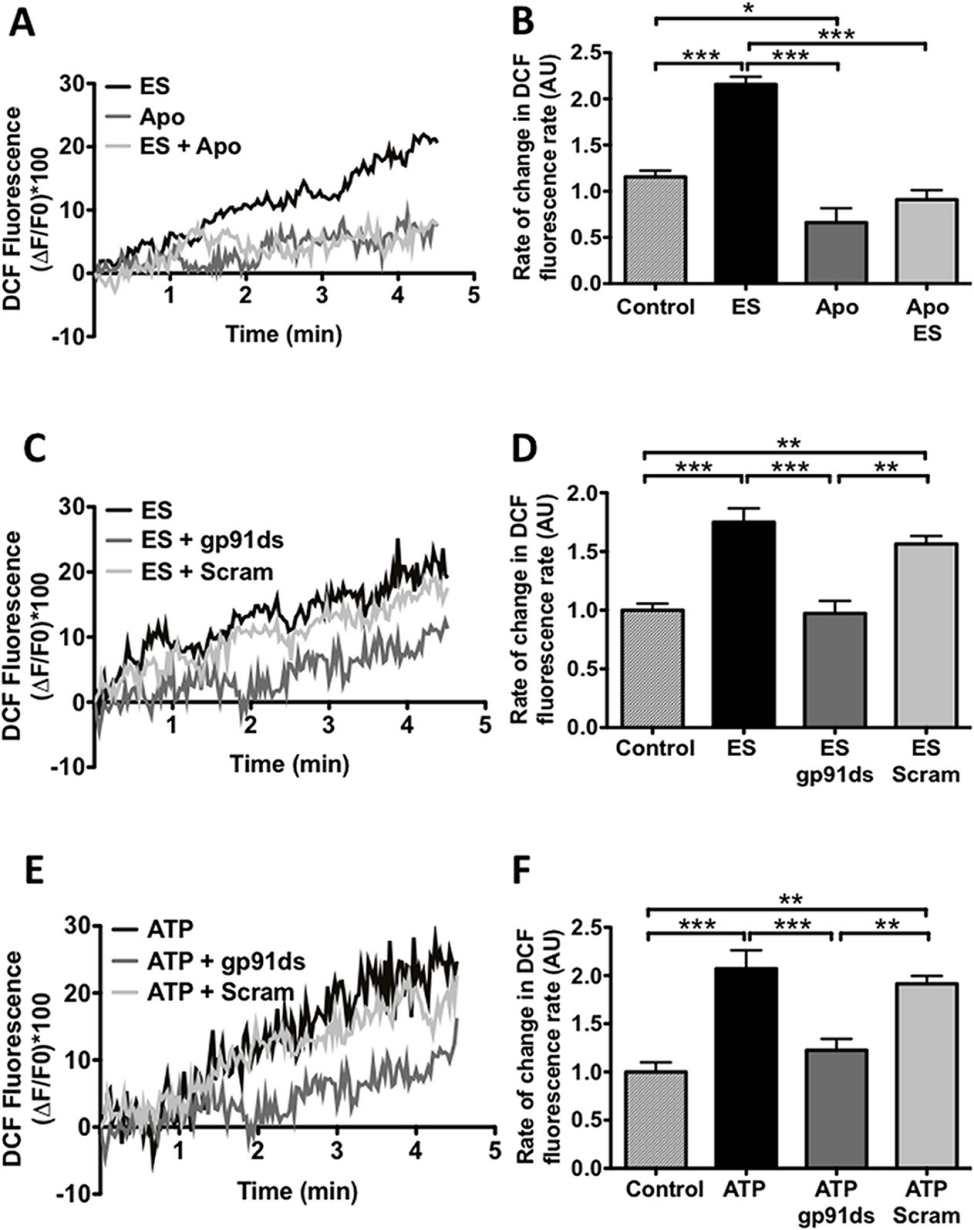
Electrical stimulation and exogenous ATP increase ROS production via NOX2. Muscle fibers were isolated, loaded with DCF (30min) and stimulated with electrical stimulation (ES) or exogenous ATP A, representative traces of DCF fluorescence under control conditions or stimulated with ES in the absence or presence of apocynin. B, Muscle cells were stimulated with ES and the slope of fluorescence was analyzed (*see* *Materials and Methods*). C, Representative traces of DCF fluorescence under control conditions or stimulated with ES in the absence or presence of gp91ds-TAT or scrambled peptide. D, muscle cells were stimulated with ES and the slope of fluorescence was analyzed (*see* *material and method*). E, representative traces of DCF fluorescence under control or stimulated with exogenous ATP in the absence or presence of gp91ds-TAT or scrambled peptide. F, muscle cells were stimulated with ES and the slope of fluorescence was analyzed (*see* *material and method*)(n = 5, *p<0.05, **p<0.01, ***p<0.001).

NOX2 is one of the main sources of ROS in skeletal muscle [8] but its activation by ATP has not been shown in adult skeletal muscle fibers. In order to study the ATP-dependent source of ROS in adult muscle, ES- dependent ROS production was studied in the presence of apocynin, a NOX2 inhibitor (Fig 4A and 4B). Apocynin completely blocked H_2_O_2_ production, but as it is a nonspecific NOX2 inhibitor [31], we used gp91 ds-tat peptide which blocks the recognition site between the NOX2 subunits [23]. ES-dependent ROS increase was inhibited by ds-tat peptide gp91 (Fig 4C and 4D) and not by the scrambled peptide used as control. In addition, exogenous ATP increased ROS production and this effect was blocked with gp91 ds-tat peptide (Fig 4E and 4F). These results strongly suggest that ROS production induced by either ES or exogenous ATP is mediated by NOX2.

### ATP-dependent ROS production via NOX2 requires PKC activation

**Fig 5:**
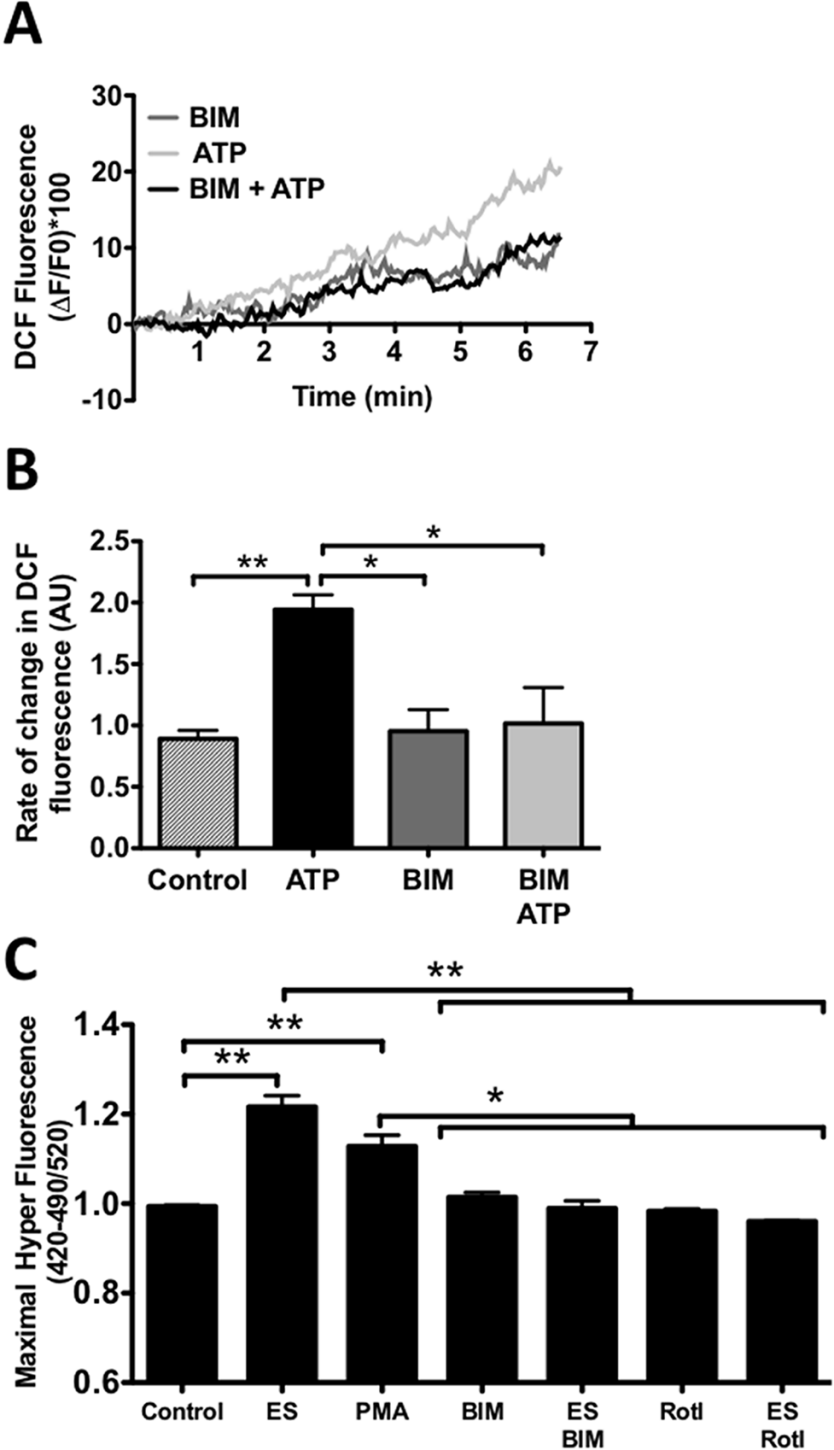
Extracellular ATP induces NOX2 activation via PKC. Muscle fibers were isolated, loaded with DCF (30min) and stimulated with exogenous ATP. A, representative traces of DCF fluorescence under control or stimulated with ATP in the absence or presence of BIM (5μM). B, muscle cells were stimulated with ATP and the slope of fluorescence was analyzed (*see* *material and method*) (n = 3, *p<0.05). C, muscle fibers were isolated and transfected with HyPer plasmid, 24h post transfection the cells were stimulated in the presence of PMA, BIM or Rotterin (Rotl) as indicated in the graph, maximal fluorescence was plotted (n = 5), *p<0.05, **p<0.01.

To study the possible role of PKC in ATP-dependent ROS generation we used the DCF probe. Incubation with BIM, a non selective general PKC inhibitor, strongly decreased the effect of ATP stimulation on ROS production (Fig 5A and 5B). In addition, in muscle fibers transfected with HyPer, BIM decreased significantly the ATP-dependent ROS increase. Similar effect was observed when using rottlerin, a PKCδ inhibitor (Fig 5C). Finally, we observed that the phorbol 12-myristate 13-acetate (PMA) mimicked the effect of exogenous ATP on ROS production. These results suggest that diacylglycerol-dependent PKCs mediate ROS production induced by extracellular ATP (Fig 5C).

## Discussion

**Fig 6:**
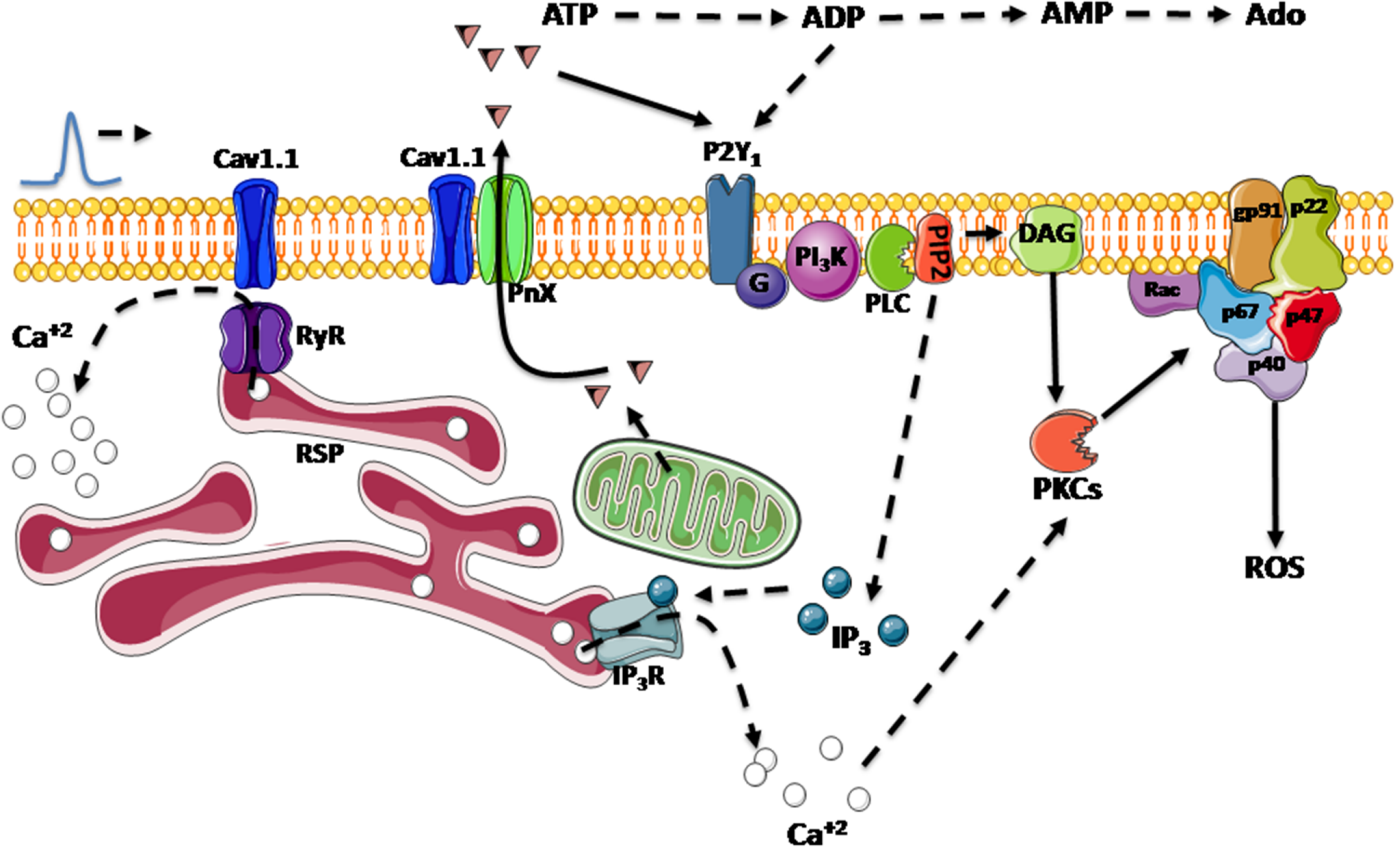
Working model. Electrical stimulation in adult FDB fibers activates Cav1.1 with each depolarizing event. This activation in turn induces ATP release via PnX1 channel. These events will trigger in turn a signaling cascade where, through activation of P2Y_1_ receptors, PI3K and PLC and consequent PKC activation induces NOX2 activation and ROS production. Dotted lines show signaling pathways already described. Solid lines show our observations.

In this work, we describe a novel mechanism for ROS production via PKC-NOX2 activation induced by extracellular ATP released from adult cultured muscle cells after electrical stimulation which mimics the physiological activation of skeletal muscle during exercise (Fig 6). Electrical stimulation is able to modulate several cascades triggered by ATP release and P2Y purinergic receptors such as glucose uptake, calcium signaling, gene expression and muscle plasticity [20, 26, 29, 32]. Interstitial ATP concentration increases during muscle contraction and this increase is intensity-dependent, possibly reaching micromolar concentrations in the T-tubule lumen [33, 34]. 10 μM was used to stimulate the muscle cells, a concentration previously reported as sufficient to induce effects on skeletal muscle cells [29]. Considering that purinergic receptors are located in the transverse tubule membrane and the presence of active ectonucleotidases in that region, it is not possible to calculate the actual ATP concentration that reaches the receptor after diffusion and degradation.

Different cell types increase ROS production upon membrane depolarization. For example, endothelial cells from human umbilical vein (HUVEC) increase ROS production after incubation with a high K^+^ concentration [35]. Previously our laboratory reported that electrical stimulation induces ROS production from NOX2 in muscle cells and this effect is Cav1.1 dependent [7]. During depolarization, muscle cells release ATP trough PnX1 channel; this process is prevented by nifedipine, a Cav1.1 blocker [26]. In addition, these proteins are located in the T-tubule and we recently showed that both proteins appear to be part of the same signaling complex [36]. We show here that electrical stimulation increases ROS production and that this increase was blocked by CBX, an inhibitor of PnX1 channel; this effect was mimicked by exogenous ATP, suggesting that ATP released via PnX1 during ES is necessary to increase ROS production during depolarization of skeletal muscle fibers.

There are several sources of ROS in skeletal muscle cells, such as mitochondria metabolism, xanthine oxidase, NOX2 and NOX4 [3]. However, the main ROS source during muscle contraction appears to be NOX2 [8]. Extracellular ATP can induce NOX2 activation in different cell models as macrophages and lung epithelial cells [37, 38]. Our study shows that ROS production induced by ES or exogenous ATP was prevented by both apocynin and gp91dsTAT, known NOX2 inhibitors. Our results suggest that ROS production induced by ES is dependent on ATP released from muscle cells via PnX1, which in turn induces NOX2 activation. These results are consistent with previous reports; for example, in myoblasts, extracellular ATP increases ROS production via NADPH oxidase, promoting cell proliferation [14].

DCF probe is sensitive to multiple types of ROS, including several reactive oxygen and nitrogen species [39]. Probably ES (via extracellular ATP) can induce oxidation of the probe by different oxidative species. For example, superoxide anion produced by NOX2 can dismutate to H_2_O_2_ in the cytoplasm via superoxide dismutase (CuZnSOD). However, superoxide can react with nitric oxide to form peroxynitrite (ONOO-). The rate of reaction to produce ONOO- is larger than the speed of enzymatic dismutation (7x10^9^ vs 2x10^9^ M^-1^s^-1^ respectively) [10]. To determine the reactive species induced by extracellular ATP, we used the protein Hyper which has high sensitivity and specificity toward hydrogen peroxide and is based on the properties of OxyR, an E. coli protein especially devoted to sensing H_2_O_2_ [25]. Extracellular ATP induced a transient H_2_O_2_ increase which was similar to that already reported by us in skeletal muscle cells depolarized with high K^+^ [24]. In addition, in fibers treated with apyrase to completely hydrolyze ATP, ES did not increase ROS production. These results suggest that extracellular ATP plays a key role on ROS production induced by ES.

Skeletal muscle cell express several purinergic receptors of the P2X and P2Y families [21]. We previously showed that the most abundant P2Y receptor subtypes in adult skeletal muscle are P2Y_1_, P2Y_2_, P2Y_4_ and P2Y_6_. Moreover, P2Y_1_ and P2Y_2_, two of the ATP/ADP-responsive receptors, are highly expressed in relation to the other subtypes [29]. In this work we provided evidence pointing to P2Y_1_ as the target for extracellular ATP to induce ROS increase. The P2Y_1_ receptor has an important role in skeletal muscle function. For example, extracellular ATP acts via P2Y_1_ receptors to stimulate acetylcholinesterase and acetylcholine receptor expression [40]. Extracellular ATP appears to inhibit chloride channels in mature mammalian skeletal muscle by activating P2Y_1_ receptors [41] and participates in up-regulation of Na/K-ATPase activity during muscle activity [42]. This receptor is G-protein (Gq) coupled and downstream increases IP_3_ production and PKC activation via PLC [43]. NOX2 can be activated by different mechanisms, including p47^phox^ subunit phosphorylation by PI3K or PKC [4]. Skeletal muscle expresses several PKC isoforms. We previously demonstrated that myotubes depolarization activate NOX2 via PKCs and that in muscle fibers insulin leads to activation of NOX2 by PKCδ [15, 44]. In this study, we showed that both BIM and rottllerin prevented ROS production in response to extracellular ATP. In addition, the increase ROS production was mimicked by PMA, an analogous of DAG. These results suggest that extracellular ATP induces NOX2 activation via P2Y_1_/PKC and that this PKC is DAG dependent, however the specific isoform of PKC involved is yet to be identified. It is important to note that all inhibitors used in this work were tested first in the absence of ATP stimulation and show no effect on basal H_2_O_2_ production, suggesting lack of non-specific effects on NOX2. We probed the effect of suramin, CBX, BIM and rottlerin, in the fluorescence increase induced by exogenous H_2_O_2_ addition (data not shown), only suramin has an antioxidant effect in itself; however the role of ATP receptor is validated using exogenous ATP and P2Y1 agonist.

Intracellular ROS may produce different effects such as mitochondria biogenesis, antioxidant enzymes expression and calcium signals [12]. Physiological regulation of cell signaling events by ROS occurs primarily via selective modification of cysteine residues within proteins [45]. Since cells possess specific systems to reverse these protein redox modifications, transient modifications of cysteine residues are likely to be central to the mechanisms underlying redox regulation of normal cell function [45]. ROS can also induce changes in intracellular calcium levels, which are the result of oxidative modification of calcium channels or other proteins involved in calcium signaling [2]. In cardiomyocytes, myotubes and isolated triads from skeletal muscle, ROS originated from NOX2 induce calcium release via ryanodine receptor (RyR) [7, 9]. It has been suggested that this ROS production may be necessary for the excitation-contraction (E-C) coupling process [9]. For example, NOX2 is located in the membrane of the t-tubules and in isolated triads, NOX2 increase calcium release through RyR oxidation [9, 46]. The RyR1 isoform [46] is essential for skeletal muscle excitation-contraction (E-C) coupling, a process that requires close physical interactions between RyR1 present in junctional SR and Cav1.1, which acts as the voltage sensor protein in T-tubules responsible for RyR1 activation during E-C coupling [47]. Despite that Sakellariou already described that NOX2 is involved in ROS generation evoked by contraction [8], our work shows new relevant data about the mechanism involved, evocating a new pathway associated to E-C coupling that depends of ATP receptor, panexins and NOX2. In heart muscle, NOX2 can induce RyR oxidation increasing calcium release from sarcoendoplasmic reticulum [27]. Calcium release during E-C is essential for muscle contraction. The privileged location of the skeletal muscle NOX2 at the T-tubules and its activation during contraction opens the possibility that conditions of increased muscle activity, such as exercise, could potentiate calcium release through NOX-induced RyR1 redox activation. Recently, Riquelme et al. [48] showed that ATP released from muscle fibers via PnX1 is required for potentiating skeletal muscle contraction and that this effect is mediated by P2Y_1_ purinergic receptors. We suggest that ES induces ATP release from muscle fibers via PnX1; extracellular ATP can activate P2Y_1_ receptors, followed by downstream PKC activation. PKC induces NOX2 activation, increasing ROS production; ROS could induce RyR1 redox activation, enhancing the channel activity (Fig 6). At least one important function influenced by ROS-dependent RyR1 activation has been described in skeletal muscle cells; insulin elicited GLUT4 translocation to the membrane depends on calcium release after H_2_O_2_ oxidation of RyR1 in L-6 cells [49]. More research is needed to ascertain whether other important functions of the muscle cell may be regulated in the same way.

## References

[1] Coffey VG, Hawley JA. The molecular bases of training adaptation. Sports medicine. 2007;37(9):737–63. . 17722947 10.2165/00007256-200737090-00001

[2] Droge W. Free radicals in the physiological control of cell function. Physiological reviews. 2002;82(1):47–95. 10.1152/physrev.00018.2001 . 11773609

[3] Jackson MJ. Control of reactive oxygen species production in contracting skeletal muscle. Antioxidants & redox signaling. 2011;15(9):2477–86. 10.1089/ars.2011.3976 21699411 PMC3176346

[4] Bedard K, Krause KH. The NOX family of ROS-generating NADPH oxidases: physiology and pathophysiology. Physiological reviews. 2007;87(1):245–313. 10.1152/physrev.00044.2005 . 17237347

[5] Inoguchi T, Sonta T, Tsubouchi H, Etoh T, Kakimoto M, Sonoda N, et al Protein kinase C-dependent increase in reactive oxygen species (ROS) production in vascular tissues of diabetes: role of vascular NAD(P)H oxidase. Journal of the American Society of Nephrology: JASN. 2003;14(8 Suppl 3):S227–32. . 12874436 10.1097/01.asn.0000077407.90309.65

[6] Sun QA, Wang B, Miyagi M, Hess DT, Stamler JS. Oxygen-coupled redox regulation of the skeletal muscle ryanodine receptor/Ca2+ release channel (RyR1): sites and nature of oxidative modification. The Journal of biological chemistry. 2013;288(32):22961–71. 10.1074/jbc.M113.480228 23798702 PMC3743473

[7] Espinosa A, Leiva A, Pena M, Muller M, Debandi A, Hidalgo C, et al Myotube depolarization generates reactive oxygen species through NAD(P)H oxidase; ROS-elicited Ca2+ stimulates ERK, CREB, early genes. Journal of cellular physiology. 2006;209(2):379–88. 10.1002/jcp.20745 . 16897752

[8] Sakellariou GK, Vasilaki A, Palomero J, Kayani A, Zibrik L, McArdle A, et al Studies of mitochondrial and nonmitochondrial sources implicate nicotinamide adenine dinucleotide phosphate oxidase(s) in the increased skeletal muscle superoxide generation that occurs during contractile activity. Antioxidants & redox signaling. 2013;18(6):603–21. 10.1089/ars.2012.4623 23050834 PMC3549212

[9] Hidalgo C, Sanchez G, Barrientos G, Aracena-Parks P. A transverse tubule NADPH oxidase activity stimulates calcium release from isolated triads via ryanodine receptor type 1 S-glutathionylation. The Journal of biological chemistry. 2006;281(36):26473–82. 10.1074/jbc.M600451200 . 16762927

[10] D'Autreaux B, Toledano MB. ROS as signalling molecules: mechanisms that generate specificity in ROS homeostasis. Nature reviews Molecular cell biology. 2007;8(10):813–24. 10.1038/nrm2256 . 17848967

[11] Flores RV, Hernandez-Perez MG, Aquino E, Garrad RC, Weisman GA, Gonzalez FA. Agonist-induced phosphorylation and desensitization of the P2Y2 nucleotide receptor. Molecular and cellular biochemistry. 2005;280(1–2):35–45. 10.1007/s11010-005-8050-5 16311903 PMC1633720

[12] Haendeler J, Tischler V, Hoffmann J, Zeiher AM, Dimmeler S. Low doses of reactive oxygen species protect endothelial cells from apoptosis by increasing thioredoxin-1 expression. FEBS letters. 2004;577(3):427–33. 10.1016/j.febslet.2004.10.041 . 15556622

[13] Scheele C, Nielsen S, Pedersen BK. ROS and myokines promote muscle adaptation to exercise. Trends in endocrinology and metabolism: TEM. 2009;20(3):95–9. 10.1016/j.tem.2008.12.002 . 19269849

[14] Sciancalepore M, Luin E, Parato G, Ren E, Giniatullin R, Fabbretti E, et al Reactive oxygen species contribute to the promotion of the ATP-mediated proliferation of mouse skeletal myoblasts. Free radical biology & medicine. 2012;53(7):1392–8. 10.1016/j.freeradbiomed.2012.08.002 . 22917975

[15] Espinosa A, Garcia A, Hartel S, Hidalgo C, Jaimovich E. NADPH oxidase and hydrogen peroxide mediate insulin-induced calcium increase in skeletal muscle cells. The Journal of biological chemistry. 2009;284(4):2568–75. 10.1074/jbc.M804249200 . 19028699

[16] Jaimovich E, Reyes R, Liberona JL, Powell JA. IP(3) receptors, IP(3) transients, and nucleus-associated Ca(2+) signals in cultured skeletal muscle. American journal of physiology Cell physiology. 2000;278(5):C998–C1010. . 10794674 10.1152/ajpcell.2000.278.5.C998

[17] Araya R, Liberona JL, Cardenas JC, Riveros N, Estrada M, Powell JA, et al Dihydropyridine receptors as voltage sensors for a depolarization-evoked, IP3R-mediated, slow calcium signal in skeletal muscle cells. The Journal of general physiology. 2003;121(1):3–16. 12508050 10.1085/jgp.20028671 PMC2217318

[18] Eltit JM, Hidalgo J, Liberona JL, Jaimovich E. Slow calcium signals after tetanic electrical stimulation in skeletal myotubes. Biophysical journal. 2004;86(5):3042–51. 10.1016/S0006-3495(04)74353-2 15111418 PMC1304170

[19] Eltit JM, Garcia AA, Hidalgo J, Liberona JL, Chiong M, Lavandero S, et al Membrane electrical activity elicits inositol 1,4,5-trisphosphate-dependent slow Ca2+ signals through a Gbetagamma/phosphatidylinositol 3-kinase gamma pathway in skeletal myotubes. The Journal of biological chemistry. 2006;281(17):12143–54. 10.1074/jbc.M511218200 . 16513646

[20] Buvinic S, Almarza G, Bustamante M, Casas M, Lopez J, Riquelme M, et al ATP released by electrical stimuli elicits calcium transients and gene expression in skeletal muscle. The Journal of biological chemistry. 2009;284(50):34490–505. 10.1074/jbc.M109.057315 19822518 PMC2787310

[21] Burnstock G, Arnett TR, Orriss IR. Purinergic signalling in the musculoskeletal system. Purinergic signalling. 2013;9(4):541–72. 10.1007/s11302-013-9381-4 23943493 PMC3889393

[22] Prosser BL, Ward CW, Lederer WJ. X-ROS signaling: rapid mechano-chemo transduction in heart. Science. 2011;333(6048):1440–5. 10.1126/science.1202768 . 21903813

[23] Rey FE, Cifuentes ME, Kiarash A, Quinn MT, Pagano PJ. Novel competitive inhibitor of NAD(P)H oxidase assembly attenuates vascular O(2)(-) and systolic blood pressure in mice. Circulation research. 2001;89(5):408–14. . 11532901 10.1161/hh1701.096037

[24] Espinosa A, Campos C, Diaz-Vegas A, Galgani JE, Juretic N, Osorio-Fuentealba C, et al Insulin-dependent H2O2 production is higher in muscle fibers of mice fed with a high-fat diet. International journal of molecular sciences. 2013;14(8):15740–54. 10.3390/ijms140815740 23899788 PMC3759883

[25] Belousov VV, Fradkov AF, Lukyanov KA, Staroverov DB, Shakhbazov KS, Terskikh AV, et al Genetically encoded fluorescent indicator for intracellular hydrogen peroxide. Nature methods. 2006;3(4):281–6. 10.1038/nmeth866 . 16554833

[26] Jorquera G, Altamirano F, Contreras-Ferrat A, Almarza G, Buvinic S, Jacquemond V, et al Cav1.1 controls frequency-dependent events regulating adult skeletal muscle plasticity. Journal of cell science. 2013;126(Pt 5):1189–98. 10.1242/jcs.116855 . 23321639

[27] Prosser BL, Khairallah RJ, Ziman AP, Ward CW, Lederer WJ. X-ROS signaling in the heart and skeletal muscle: stretch-dependent local ROS regulates [Ca(2)(+)]i. Journal of molecular and cellular cardiology. 2013;58:172–81. 10.1016/j.yjmcc.2012.11.011 23220288 PMC3951390

[28] Khairallah RJ, Shi G, Sbrana F, Prosser BL, Borroto C, Mazaitis MJ, et al Microtubules underlie dysfunction in duchenne muscular dystrophy. Science signaling. 2012;5(236):ra56 10.1126/scisignal.2002829 22871609 PMC3835660

[29] Fernandez-Verdejo R, Casas M, Galgani JE, Jaimovich E, Buvinic S. Exercise sensitizes skeletal muscle to extracellular ATP for IL-6 expression in mice. International journal of sports medicine. 2014;35(4):273–9. 10.1055/s-0033-1353147 . 24022572

[30] Abbracchio MP, Burnstock G, Boeynaems JM, Barnard EA, Boyer JL, Kennedy C, et al International Union of Pharmacology LVIII: update on the P2Y G protein-coupled nucleotide receptors: from molecular mechanisms and pathophysiology to therapy. Pharmacological reviews. 2006;58(3):281–341. 10.1124/pr.58.3.3 16968944 PMC3471216

[31] Vejrazka M, Micek R, Stipek S. Apocynin inhibits NADPH oxidase in phagocytes but stimulates ROS production in non-phagocytic cells. Biochimica et biophysica acta. 2005;1722(2):143–7. 10.1016/j.bbagen.2004.12.008 . 15716123

[32] Osorio-Fuentealba C, Contreras-Ferrat AE, Altamirano F, Espinosa A, Li Q, Niu W, et al Electrical stimuli release ATP to increase GLUT4 translocation and glucose uptake via PI3Kgamma-Akt-AS160 in skeletal muscle cells. Diabetes. 2013;62(5):1519–26. 10.2337/db12-1066 23274898 PMC3636621

[33] Mortensen SP, Gonzalez-Alonso J, Nielsen JJ, Saltin B, Hellsten Y. Muscle interstitial ATP and norepinephrine concentrations in the human leg during exercise and ATP infusion. Journal of applied physiology. 2009;107(6):1757–62. 10.1152/japplphysiol.00638.2009 . 19797688

[34] Hellsten Y, Maclean D, Radegran G, Saltin B, Bangsbo J. Adenosine concentrations in the interstitium of resting and contracting human skeletal muscle. Circulation. 1998;98(1):6–8. . 9665052 10.1161/01.cir.98.1.6

[35] Sohn HY, Keller M, Gloe T, Morawietz H, Rueckschloss U, Pohl U. The small G-protein Rac mediates depolarization-induced superoxide formation in human endothelial cells. The Journal of biological chemistry. 2000;275(25):18745–50. 10.1074/jbc.M000026200 . 10764736

[36] Jorquera G, Juretic N, Jaimovich E, Riveros N. Membrane depolarization induces calcium-dependent upregulation of Hsp70 and Hmox-1 in skeletal muscle cells. American journal of physiology Cell physiology. 2009;297(3):C581–90. 10.1152/ajpcell.00167.2009 . 19570893

[37] Cruz CM, Rinna A, Forman HJ, Ventura AL, Persechini PM, Ojcius DM. ATP activates a reactive oxygen species-dependent oxidative stress response and secretion of proinflammatory cytokines in macrophages. The Journal of biological chemistry. 2007;282(5):2871–9. 10.1074/jbc.M608083200 17132626 PMC2693903

[38] Cheng SE, Lee IT, Lin CC, Wu WL, Hsiao LD, Yang CM. ATP mediates NADPH oxidase/ROS generation and COX-2/PGE2 expression in A549 cells: role of P2 receptor-dependent STAT3 activation. PloS one. 2013;8(1):e54125 10.1371/journal.pone.0054125 23326583 PMC3543320

[39] Crow JP. Dichlorodihydrofluorescein and dihydrorhodamine 123 are sensitive indicators of peroxynitrite in vitro: implications for intracellular measurement of reactive nitrogen and oxygen species. Nitric oxide: biology and chemistry / official journal of the Nitric Oxide Society. 1997;1(2):145–57. 10.1006/niox.1996.0113 . 9701053

[40] Choi RC, Siow NL, Cheng AW, Ling KK, Tung EK, Simon J, et al ATP acts via P2Y1 receptors to stimulate acetylcholinesterase and acetylcholine receptor expression: transduction and transcription control. The Journal of neuroscience: the official journal of the Society for Neuroscience. 2003;23(11):4445–56. . 12805285 10.1523/JNEUROSCI.23-11-04445.2003 PMC6740789

[41] Voss AA. Extracellular ATP inhibits chloride channels in mature mammalian skeletal muscle by activating P2Y1 receptors. The Journal of physiology. 2009;587(Pt 23):5739–52. 10.1113/jphysiol.2009.179275 19805741 PMC2805382

[42] Walas H, Juel C. Purinergic activation of rat skeletal muscle membranes increases Vmax and Na+ affinity of the Na,K-ATPase and phosphorylates phospholemman and alpha1 subunits. Pflugers Archiv: European journal of physiology. 2012;463(2):319–26. 10.1007/s00424-011-1050-2 . 22057585

[43] N DA, Volonte C. Metabotropic purinergic receptors in lipid membrane microdomains. Current medicinal chemistry. 2013;20(1):56–63. . 23151003

[44] Fill M, Copello JA. Ryanodine receptor calcium release channels. Physiological reviews. 2002;82(4):893–922. 10.1152/physrev.00013.2002 . 12270947

[45] Powers SK, Duarte J, Kavazis AN, Talbert EE. Reactive oxygen species are signalling molecules for skeletal muscle adaptation. Experimental physiology. 2010;95(1):1–9. 10.1113/expphysiol.2009.050526 19880534 PMC2906150

[46] Hidalgo C, Donoso P, Carrasco MA. The ryanodine receptors Ca2+ release channels: cellular redox sensors? IUBMB life. 2005;57(4–5):315–22. 10.1080/15216540500092328 . 16036616

[47] Rios E, Pizarro G. Voltage sensor of excitation-contraction coupling in skeletal muscle. Physiological reviews. 1991;71(3):849–908. . 2057528 10.1152/physrev.1991.71.3.849

[48] Riquelme MA, Cea LA, Vega JL, Boric MP, Monyer H, Bennett MV, et al The ATP required for potentiation of skeletal muscle contraction is released via pannexin hemichannels. Neuropharmacology. 2013;75:594–603. 10.1016/j.neuropharm.2013.03.022 . 23583931

[49] Contreras-Ferrat A, Llanos P, Vasquez C, Espinosa A, Osorio-Fuentealba C, Arias-Calderon M, et al Insulin elicits a ROS-activated and an IP(3)-dependent Ca(2)(+) release, which both impinge on GLUT4 translocation. Journal of cell science. 2014;127(Pt 9):1911–23. 10.1242/jcs.138982 . 24569874

